# ITNCountry: Transportation for Rural and Small Communities

**DOI:** 10.1093/geroni/igaf122.1291

**Published:** 2025-12-31

**Authors:** Katherine Freund, Aaron Meuser

**Affiliations:** ITNAmerica, Westbrook, Maine, United States; ITN America, Westbrook, Maine, United States

## Abstract

Providing transportation in rural and small communities presents unique challenges -- resources diminish as distances and costs increase, while efficiency declines. This research demonstrates ITNCountry on the Salesforce platform in small communities in California, Maine, Kentucky, Florida, and New York to address these challenges. The demonstration occurred in two phases. Phase One: (1) rebuild ITNRides software on Salesforce; (2) develop marketing strategies; and (3) collaborate with ITN affiliates to establish ITNCountry in small and rural communities. Phase Two: (1) transition demonstration communities to ITNRides2.0; (2) train communities in ride services and coordination; (3) utilize the Online Learning Center for training; and (4) market and implement innovative payment programs. Data were collected over 12 months, with an additional 8 months as service continued. Methods included community profiles, stakeholder interviews, and pre- and post-surveys of stakeholders, riders, and volunteer drivers. Rides increased steadily, and rider and volunteer surveys showed high satisfaction. Over 60% of rides were for healthcare, 44% of riders used a mobility aid and 29% required door-through-door assistance. No membership was required, but survey results indicated that riders valued membership for its sense of belonging, community and security. ITNCountry volunteer transportation in rural and small communities shows promise in meeting the mobility needs of older adults and people with mobility challenges.

